# Calcium signaling in plant mineral nutrition: From uptake to transport

**DOI:** 10.1016/j.xplc.2023.100678

**Published:** 2023-08-26

**Authors:** Tian Wang, Xuanyi Chen, Chuanfeng Ju, Cun Wang

**Affiliations:** 1 National Key Laboratory of Crop Improvement for Stress Tolerance and Production, College of Life Sciences, Northwest Agriculture & Forestry University, Yangling, Shaanxi 712100, China

**Keywords:** Ca^2+^ signaling, mineral nutrition, ion channels and transporters, uptake and transport

## Abstract

Plant mineral nutrition is essential for crop yields and human health. However, the uneven distribution of mineral elements over time and space leads to a lack or excess of available mineral elements in plants. Among the essential nutrients, calcium (Ca^2+^) stands out as a prominent second messenger that plays crucial roles in response to extracellular stimuli in all eukaryotes. Distinct Ca^2+^ signatures with unique parameters are induced by different stresses and deciphered by various Ca^2+^ sensors. Recent research on the participation of Ca^2+^ signaling in regulation of mineral elements has made great progress. In this review, we focus on the impact of Ca^2+^ signaling on plant mineral uptake and detoxification. Specifically, we emphasize the significance of Ca^2+^ signaling for regulation of plant mineral nutrition and delve into key points and novel avenues for future investigations, aiming to offer new insights into plant ion homeostasis.

## Teaser

This review provides a comprehensive overview of Ca^2+^ signaling in plant mineral uptake and detoxification. The role of Ca^2+^ as a second messenger in response to extracellular stimuli and the distinct Ca^2+ signatures induced by different stresses are^ highlighted. The review also offers insights into future investigations of plant ion homeostasis.

## Introduction

Plant growth and development are affected by changing environmental conditions and various stress factors, including soil mineral content and accumulation of harmful elements. The soil provides plants with 14 essential mineral nutrients, which are categorized into macronutrients and micronutrients on the basis of their dry biomass percentage (< or ≥ 0.1%). Macronutrients consist of nitrogen (N), phosphorus (P), potassium (K), sulfur (S), calcium (Ca), and magnesium (Mg), and micronutrients include iron (Fe), manganese, copper, zinc, molybdenum, boron (B), chloride, and nickel (Maathuis and Diatloff, 2013; Vatansever et al., 2017). Besides the essential elements, some elements in the soil are not essential for plant growth and reproduction but are conducive to plant growth; these include sodium, silicon, cobalt, and selenium (Pilon-Smits et al., 2009; Vatansever et al., 2017). However, a class of trace metals or metalloid elements, including cadmium, lead, chromium, arsenic (As), and aluminum, can be severely toxic to plants (Ghori et al., 2019).

Ca^2+^ is an essential nutrient and the most prominent second messenger, playing a crucial role in response to extracellular stimuli in all eukaryotes (Lee and Seo, 2021). Different stresses cause distinct Ca^2+^ signatures (also called stimulus-specific Ca^2+^ patterns): Ca^2+^ signals with different parameters such as transient or repetitive oscillation, duration, amplitude, frequency, and spatial distribution (Kudla et al., 2018b). Ca^2+^ influx via Ca^2+^ channels and Ca^2+^ efflux via Ca^2+^ transporters comprise an orchestrated balanced system. Ca^2+^ channels include cyclic nucleotide-gated channels (CNGCs), glutamate receptor-like channels, the mechanosensitive channel of small conductance (MscS)-like channels, *Mid1*-complementing activity channels, reduced hyperosmolality-induced [Ca^2+^]_i_ increase (hyperosmolality-gated calcium-permeable channels [OSCA]) channels, two-pore channels, annexins, and MILDEW RESISTANCE LOCUS O proteins (Kudla et al., 2018a; Luan and Wang, 2021; Guichard et al., 2022; Gao et al., 2023). Ca^2+^ transporters include Ca^2+^-ATPases and Ca^2+^/H^+^ exchangers (CAXs). Ca^2+^-ATPases are classified into two groups: P2A type (endoplasmic reticulum [ER]-type Ca^2+^-ATPase: ECA1–ECA4) and P2B type (autoinhibited Ca^2+^-ATPases: ACA1, ACA2, ACA4, and ACA7–ACA13) (García Bossi et al., 2019). CAX genes are identified as CAX1–CAX6 (Shigaki et al., 2006). The protagonists involved in the process of decoding Ca signals are Ca^2+^-binding proteins that act as sensors. They bind to Ca^2+^ in response to elevated Ca^2+^ levels in the cell and include calcineurin B-like proteins (CBLs), calmodulins (CaMs), CaM-like proteins (CMLs), Ca-dependent protein kinases (CPKs), and Ca- and CaM-dependent protein kinases. The roles of Ca^2+^ sensors and Ca^2+^ channels in Ca^2+^ signaling have been studied extensively over the past few decades and have recently been described in detail (Tian et al., 2020; Luan and Wang, 2021; Dong et al., 2022a). Here, we focused on the role of Ca^2+^ signaling in regulating plant mineral uptake and transport.

### Ca^2+^ signaling in absorption and utilization of macronutrients

#### Ca^2+^ signaling modulates N-regulatory networks

Nitrate (NO_3_^−^) serves not only as an N source but also as a signaling molecule that regulates numerous processes, including gene expression, root architecture, shoot development, seed germination, and flowering (Lin and Tsay, 2017; O'Brien et al., 2016). Several players in the NO_3^−^_ signaling pathway have been identified, including NO_3^−^_ sensors, NO_3^−^_ transporters, Ca signaling components, protein kinases, and transcription factors.

**Figure 1:**
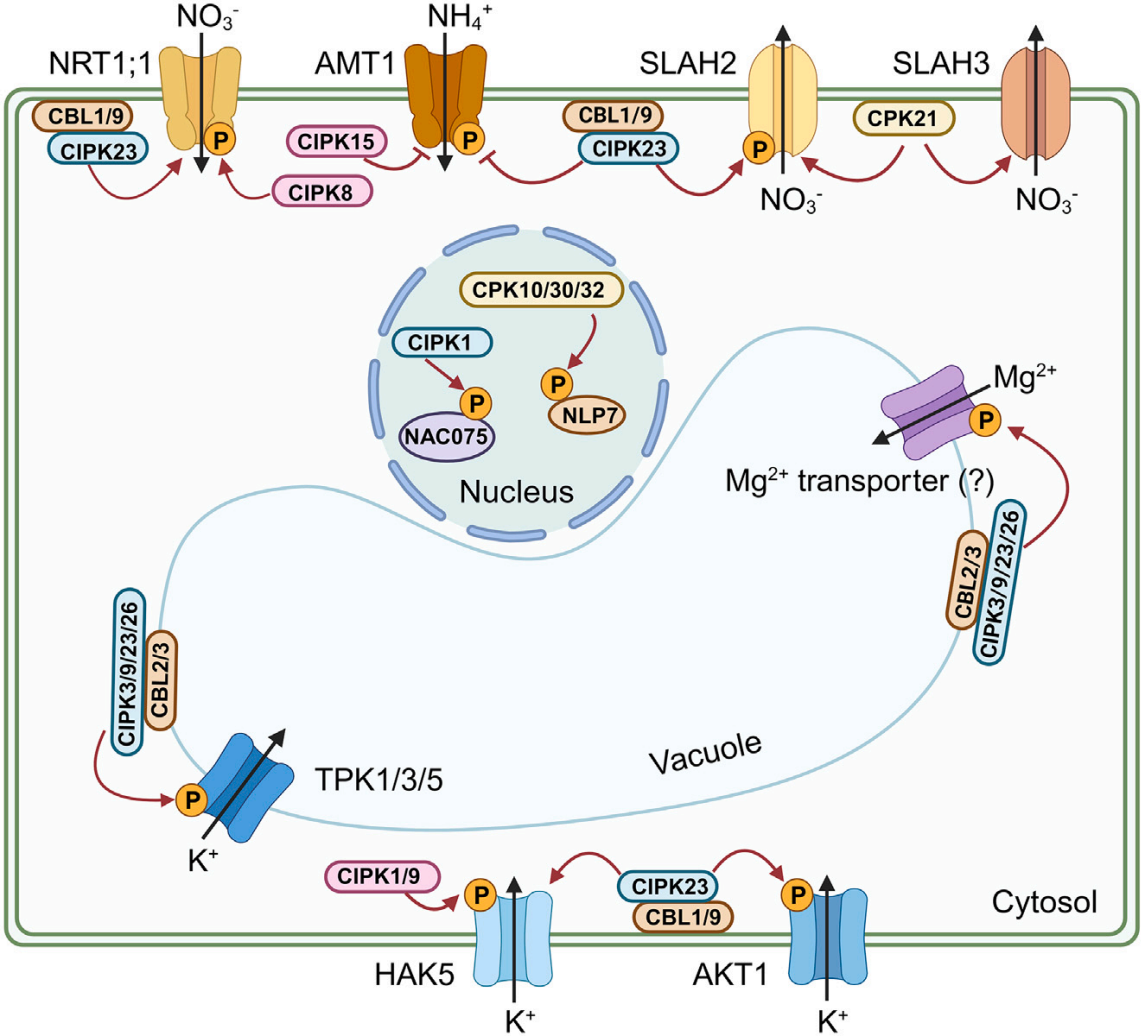
Ca^2+^ signaling regulatory network in macronutrient absorption and utilization. Under low-nitrogen (N) conditions, the CBL1/9–CIPK23 module phosphorylates NRT1;1 to enhance NO_3^−^_ uptake. Conversely, under high-N conditions, CIPK8 phosphorylates NRT1;1, facilitating NO_3_^−^ absorption. In addition, CBL1/9–CIPK23 and CIPK15 can phosphorylate AMT1, inhibiting its ammonium (NH_4_^+^) transport function and mitigating NH_4_^+^ toxicity. SLAH2 can be phosphorylated by CBL1/9–CIPK23, promoting NO_3_^−^ uptake, and CPK21 can also phosphorylate SLAH2/3 to promote NO_3_^−^ transport. NLP7, another NO_3_^−^ sensor, undergoes phosphorylation by CPK10, CPK30, and CPK32 to regulate its nucleoplasmic shuttle. CIPK1 regulates expression of downstream target genes by phosphorylating and activating NAC075 under low-N conditions. Regarding potassium (K) transport, CBL1/9 recruit their interacting kinase CIPK23 to the root cell PM. CIPK23 then phosphorylates the K transporters AKT1 and HAK5, promoting K^+^ absorption. CIPK1 and CIPK9 regulate root K uptake by phosphorylating HAK5. Under low-K^+^ conditions, the CBL2/3–CIPK3/9/23/26 module activates the tandem-pore K^+^ channels TPK1/3/5 on the tonoplast, releasing vacuolar K^+^ into the cytoplasm. The CBL2/3–CIPK3/9/23/26 module recruits magnesium (Mg^2+^) to the tonoplast and regulates downstream target transporters that mediate efficient sequestration of Mg^2+^ in vacuoles, maintaining a non-toxic level of Mg^2+^ in the cytoplasm. CBL, calcineurin B-like protein; CIPK, CBL-interacting protein kinase; NRT, NO_3^−^_ transporter; AMT1, NH_4^+^_ transporter 1; SLAH, homolog of slow type anion channel-associated 1 (SLAC1); NLP7, NIN-LIKE PROTEIN 7; CPK, Ca-dependent protein kinase; NAC075, NAC transcription factor; AKT, K^+^ transporter; HAK5, high-affinity K^+^ transporter 5; TPK, two-pore K^+^ channel.

Ca^2+^ signaling is involved in regulation of various plant growth and developmental processes, including regulation of N. NO_3^−^_ treatments rapidly and transiently increase [Ca^2+^]_cyt_ in *Arabidopsis* roots (Riveras et al., 2015). Distinct Ca^2+^ dynamics are triggered by NO_3^−^_ in the tip, pericycle, and stele of intact roots. Likewise, a gradual increase in subcellular Ca^2+^ levels attributed to NO_3^−^_ can be observed over several minutes using GCaMP6-based imaging in leaf cells co-expressing the highly sensitive Ca^2+^ biosensor GCaMP6 and a nuclear mCherry marker (Liu et al., 2017). The NITRATE TRANSPORTER 1 (NRT1) family member NRT1;1, also known as CHLORATE-RESISTANT 1 (CHL1), acts as a NO_3^−^_ sensor to perceive external NO_3^−^_ (Tsay et al., 1993; Liu et al., 1999; Remans et al., 2006). Importantly, complexes of CNGC15 and NRT1;1 dynamically regulate Ca^2+^ channel activity by sensing the external NO_3_^−^ concentration (Wang et al., 2021a). Two CBL-INTERACTING PROTEIN KINASES (CIPKs), CIPK8 and CIPK23, have been found to differentially regulate NO_3^−^_ signaling. CIPK8 plays a positive role in NO_3^−^_-induced expression of primary NO_3^−^_ response genes and acts as a positive regulator of the low-affinity response. On the other hand, CIPK23 serves as a negative regulator of the high-affinity response. In the presence of low NO_3^−^_ concentrations, CHL1 binds to NO_3^−^_ and directly interacts with CIPK23, leading to phosphorylation of CHL1 at Thr101, thereby maintaining a low-level primary response (Ho et al., 2009; Hu et al., 2009; Figure 1). Dynamic regulation of the dual-affinity system by Ca^2+^ signaling enables CHL1 to sense a wide range of NO_3^−^_ concentrations in plants and trigger different responses. Furthermore, expression of the NRT2 family members NRT2;4 and NRT2;5 is inhibited in the *cbl7* mutant under N starvation stress, indicating that CBL7 may be involved in modulation of high-affinity NO_3^−^_ uptake under NO_3_^−^ starvation conditions (Ma et al., 2015). In the plant NO_3^−^_ transport system, SLAC-ASSOCIATED 1 HOMOLOG2 (SLAH2), a homolog of slow-type anion channel-associated 1 (SLAC1), can be phosphorylated by the CBL1/9–CIPK23 module, improving its NO_3_^−^ transport function, and CPK21 can also activate SLAH2/3 to promote their NO_3_^−^ transport (Maierhofer et al., 2014; Cubero-Font et al., 2016).

To transmit N signals to intracellular and downstream signaling molecules, intracellular signal-sensing mechanisms are also required. NIN-LIKE PROTEIN 7 (NLP7) has been proposed to act as a ligand-dependent transcriptional activator and an intracellular NO_3^−^_ sensor (Alvarez et al., 2020; Liu et al., 2022). The subgroup III Ca^2+^-sensor protein kinases CPK10, CPK30, and CPK32 have been found to affect nucleoplasmic localization of NLP7 by phosphorylating its Ser205 residue (Liu et al., 2017). Two types of NO_3^−^_ sensors in the plasma membrane (PM) and cytoplasm ensure that N signals are transmitted rapidly and respond in a timely manner to different N concentrations. Expression of the basic region/leucine zipper motif (bZIP) transcription factor family members *TGA1* and *TGA4* is upregulated in a Ca^2+^-dependent manner and regulates expression of *NRT2*;*1* and *NRT2*;*2*, which mediate NO_3^−^_ transport (Alvarez et al., 2014; Zhong et al., 2015). Under low-NO_3_^−^ conditions, CIPK1 is activated and phosphorylates the NAC (NAM/ATAF/CUC) transcription factor NAC075, regulating expression of the downstream target WRKY53 (Xiao et al., 2022) (Figure 1). A series of transcription factors, including NLP7, are regulated by Ca^2+^ signaling, forming an elaborate regulatory network.

Ammonium (NH_4_^+^) is the primary source of N in many species, but excessive NH_4_^+^ can lead to NH_4^+^_ toxicity (Loqué and von Wirén, 2004). To prevent NH_4^+^_ toxicity, two members of the NH_4^+^_ transport (AMT) family, AMT1;1 and AMT1;2, are inhibited by the CBL1–CIPK23 complex (Straub et al., 2017). Expression of CIPK23 is upregulated by STOP1 when NH_4_^+^ is present in excess (Tian et al., 2021). Dynamic regulation of NRTs and AMTs by Ca^2+^ signaling components, such as the CBL1/9–CIPK23 module, helps to maintain the balance between N absorption and NH_4_^+^ toxicity. In addition, CIPK15 inhibits the activity of AMT1 isoforms by phosphorylating their C terminus (Chen et al., 2020; Figure 1). However, whether Ca^2+^ channels and other CBL–CIPK or CPK proteins are involved in regulation of AMTs requires further investigation.

### Ca^2+^ signaling regulates the response to P deficiency

P, an essential mineral nutrient for plant growth and development, is a critical component of many metabolites and macromolecules, including proteins, phospholipids, and nucleic acids (Lopez-Arredondo et al., 2014). Previous studies have demonstrated a correlation between cytosolic Ca and phosphate levels in plants. Phosphate (Pi) deficiency induces a rapid decrease in [Ca^2+^]_cyt_ in *Arabidopsis* roots (Matthus et al., 2019b, 2020) (Figure 1). A recent study found that At1g62420 (RXR3) reduces root hair growth by encoding tip-focused [Ca^2+^]_cyt_ oscillations through ROOT HAIR DEFECTIVE 6-LIKE 4 interaction with CaM under low-Pi stress (Ying and Scheible, 2022). CAX1, a vacuolar Ca^2+^/H^+^ transporter, is required for systemic Pi homeostasis involving shoot-to-root signaling in *Arabidopsis* (Liu et al., 2011). However, further investigation is necessary to determine whether Ca signaling is directly involved in regulation of P transporters and to characterize its specific regulatory mechanism. It would be interesting to investigate how Ca^2+^ channels generate Ca^2+^ signals under varying P concentrations and to determine whether CBL–CIPK modules and CPKs directly participate in regulating P signaling networks. In addition, it would be worthwhile to examine the impact of P availability on the expression and activity of Ca^2+^ channels, as well as the potential crosstalk between Ca and P signaling pathways.

### Ca^2+^ signaling adjusts K homeostasis

K is present as a soluble ion (K^+^) in plants, where it plays essential roles in many physiological processes, such as osmotic balance, stomatal regulation, protein biosynthesis, water and nutrient absorption, and enzyme activation (Wang et al., 2021b).

Studies have shown that K^+^ deficiency triggers two successive and distinct Ca^2+^ signals in roots, which exhibit spatial and temporal specificity. Ca^2+^ channels located in the root epidermis and root hair zone can be activated by hyperpolarization of the PM under K^+^ deficiency conditions (Véry and Davies, 2000; Demidchik et al., 2002). Moreover, the increase in reactive oxygen species levels induced by K^+^ deficiency can lead to Ca^2+^ signaling via reactive oxygen species–activated Ca^2+^ channels (Shin and Schachtman, 2004; Demidchik and Maathuis, 2007). The CBL–CIPK network plays a vital role in the K^+^ deficiency response. K^+^ transporter 1 (AKT1) and high-affinity K^+^ transporter 5 (HAK5), a K^+^/H^+^ symporter, are considered to be the major components involved in K^+^ uptake in *Arabidopsis* root cells under low-K^+^ conditions (Nieves-Cordones et al., 2014). CBL1/9 recruit their interacting kinase CIPK23 to the root cell PM, and CIPK23 then phosphorylates AKT1 and HAK5 to promote plant K^+^ uptake (Li et al., 2006; Xu et al., 2006; Cheong et al., 2007; Ragel et al., 2015; Lara et al., 2020). In addition, CBL10 negatively modulates AKT1 activity by competing for binding of CIPK23 to AKT1 (Ren et al., 2013). In addition to increasing their K^+^ uptake, plant cells mobilize K reserves in the vacuoles. Under low-K^+^ stress, the CBL2/3–CIPK3/9/23/26 module activates the tandem-pore K^+^ channels TPK1/3/5 on the vacuolar membrane (VM), releasing vacuolar K^+^ into the cytoplasm (Tang et al., 2020a). The PM–CBL1/9–CIPK23 and VM–CBL2/3–CIPK3/9/23/26 signaling modules play a crucial role in connecting low-K^+^ stress with activation of K^+^ channels, thereby maintaining K^+^ homeostasis. Recent studies have revealed that early occurrence of K^+^-induced activation of the vacuolar Ca^2+^ sensors CBL2/3 contributes to activation of the CBL1/9 pathway under K^+^ deficiency. A recent study showed that the protein abundance and phosphorylation status of CBL–CIPK–channel modules are influenced by external K^+^ status (Li et al., 2023b), providing unique insights into the coordinated regulation of K^+^ homeostasis by VM and PM CBL–CIPK–channel modules. Furthermore, CIPK1 and CIPK9 regulate root K^+^ uptake by phosphorylating HAK5 (Lara et al., 2020). Raf-like mitogen-activated protein kinase kinase (MAPKK) kinase (ILK1) directly interacts with HAK5 in conjunction with CML9, promoting HAK5 accumulation on the PM (Brauer et al., 2016). Together with CIPK6, CBL4 regulates the activity and PM targeting of the K^+^ channel AKT2 in a kinase interaction–dependent manner (Held et al., 2011; Figure 1). Ca^2+^ signaling has been found to participate in regulating the different localizations of K^+^ channels and transporters in different physiological processes. However, further investigations are needed to explore the potential roles of other Ca^2+^ sensors, such as CPK, in regulating K homeostasis.

### Ca^2+^ signaling and Ca nutrition

Ca is essential for plant growth and development under non-stressed and adverse conditions. Ca^2+^ not only acts as an important structural component to maintain cell wall stiffness and cell membrane stability but also plays a key role as a Ca^2+^ signal in many physiological processes, such as development and stress response (Hepler, 2005).

High levels of Ca^2+^ are harmful to plant cells (Li et al., 2014). When cytoplasmic Ca^2+^ levels become excessive, proteins such as CAX and ACAs localize to the PM or tonoplast and decrease the cytoplasmic Ca^2+^ concentration by exporting excess Ca^2+^ to the apoplast or vacuolar lumen. Furthermore, Ca^2+^ channels for influx and pumps or antiporters for efflux produce Ca^2+^ oscillators (Harper, 2001). CNGC2-mediated Ca influx and tonoplast-localized CAX1/3 jointly regulate the distribution of Ca^2+^ in plant cells, preventing excessive accumulation of Ca^2+^ in the cytoplasm and apoplastic space (Wang et al., 2017). CNGC is regulated by Ca^2+^, CaM, and regulatory motifs that bind to CaM in the CAX promoter (Martins et al., 2017). This suggests that Ca^2+^ signaling is also involved in sensing and regulation of Ca^2+^ as a nutrient. However, research in this field may be hindered by the vast majority of Ca^2+^ sensors that are typically present in cells but have little relevance to sensing of Ca^2+^ as a nutrient.

### Mg transport requires the involvement of Ca^2+^ signaling

Mg is an essential plant nutrient and a cofactor for many enzymes. It is also involved in photosynthesis and synthesis of nucleic acids and proteins. Deficiency and excess of Mg^2+^ in the soil can adversely affect plant growth and crop yields (Verbruggen and Hermans, 2013).

Ca^2+^ signaling plays a critical role in regulating the dynamic homeostasis of Mg^2+^. The CBL2/3–CIPK3/9/23/26 complex recruits Mg^2+^ to the tonoplast and further regulates downstream target transporters that mediate efficient sequestration of Mg^2+^ in vacuoles, thereby maintaining a non-toxic level of Mg^2+^ in the cytoplasm (Tang et al., 2015; Figure 1). However, the specific Mg^2+^ transporters regulated by the CBL–CIPK module remain unclear. Future efforts will focus on outstanding questions regarding the generation of specific Ca^2+^ signals in plant cells during high/low Mg^2+^ stress, clarifying the regulation mechanism of Ca^2+^ signaling on the PM and other membrane systems and identifying unknown downstream transporters or channels.

### Ca^2+^ signaling in the uptake of micronutrients

#### Ca^2+^ signals under Fe deficiency

Fe is an essential micronutrient for all organisms and an important regulator of various cellular processes involved in intracellular respiration, photosynthesis, and many other functions, such as DNA synthesis and N fixation (Vert et al., 2002).

Fe deficiency has been reported to elicit an increase in [Ca^2+^]_cyt_ in the elongation and root-hair zone, which is the main region for Fe mobilization and absorption (Tian et al., 2016). When plants are challenged with different Fe and Pi availabilities, Ca^2+^ signals also show different characteristics (Matthus et al., 2019a, 2019b). The characteristic Ca^2+^ signals detected upon external application of purine nucleotides under sufficient Fe and Pi conditions are significantly altered when plants experience Pi starvation and are restored after Fe exclusion (Matthus et al., 2019b). Under sufficient Pi conditions, Fe deficiency leads to a third, different Ca characteristic (Matthus et al., 2019a).

**Figure 2:**
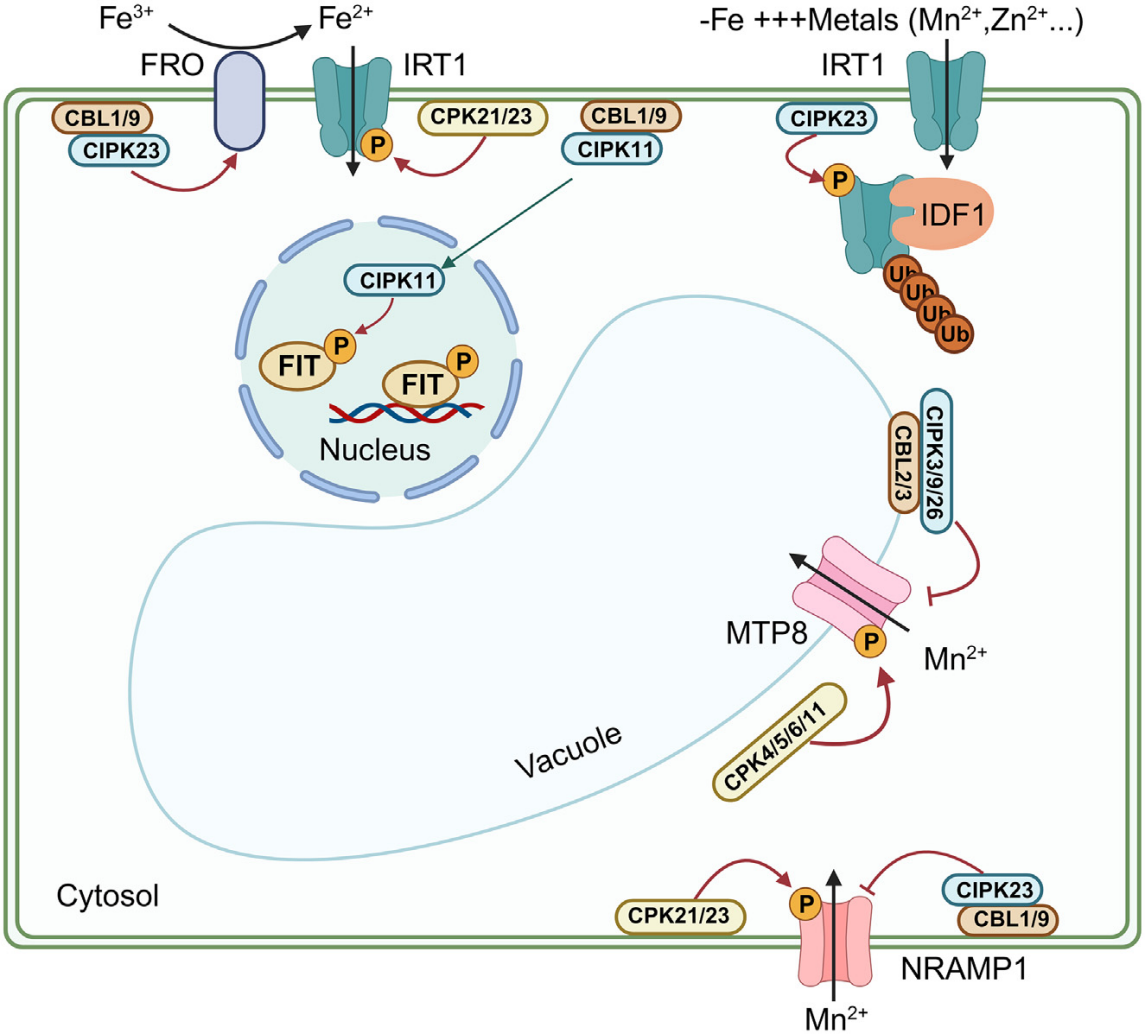
Regulation of Ca^2+^ signaling in plant uptake and transport of micronutrients. An increase in [Ca^2+^]_cyt_ induces the CBL1/9–CIPK23 module to enhance the activity of FRO, which is essential for conversion of soil Fe^3+^ into the transportable form Fe^2+^. Constitutively active CPK21 and CPK23 enhance plant tolerance to Fe deficiency by interacting with and phosphorylating IRT1. Under Fe deficiency, Ca^2+–^CBL1/9–CIPK11 phosphorylates the basic-helix-loop-helix transcription factor FIT, converting it from an inactive form to an active form. This promotes expression of downstream Fe-responsive genes and increases Fe absorption. The Fe deficiency–induced Ca^2+^ signature activates CIPK23 to phosphorylate IRT1 at the S/T residues to facilitate recruitment of IRT1 degradation factor 1 (IDF1) E3 Ub ligase in the presence of excess non-Fe metals, preventing IRT1 from transporting non-Fe metals, such as Zn and Mn. Phosphorylated IRT1 then transports other bivalent metals, such as Mn^2+^ and Zn^2+^. Four activated Ca sensors (CPK4, CPK5, CPK6, and CPK11) interact with MTP8 and phosphorylate its Ser31/32 residues, facilitating transport of excess Mn^2+^ into vacuoles. CBL2/3 recruit CIPK3/9/26 to form a complex that phosphorylates MTP8. This ultimately inhibits its activity and functions as a braking mechanism. Under Mn-deficient conditions, CPK21 and CPK23 interact with and phosphorylate NRAMP1 to enhance its transport activity. Conversely, under high Mn stress, the CBL1/9–CIPK23 complex senses Ca^2+^ signals and phosphorylates NRAMP1 at the Ser20/22 residues, triggering clathrin-mediated endocytosis of NRAMP1 and reducing Mn absorption. FRO, ferric chelate reductase; FIT, fer-like Fe deficiency-induced transcription factor; IRT1, Fe-regulated transporter 1; MTP8, metal tolerance protein 8; NRAMP1, natural resistance-associated macrophage protein 1.

Fe(III) in the soil is reduced to Fe(II), which can be absorbed and utilized by plants, via Fe(III) chelate reductase (FRO) (Khan et al., 2019). Under Fe-deficient conditions, Fe-regulated transporter 1 (IRT1) is responsible for absorption of Fe(II), while ENHANCED BENDING 1, as an Ca^2+^-dependent inhibitor, prevents Fe absorption by binding to IRT1 (Khan et al., 2019). A recent study found that constitutively active CPK21 and CPK23 enhance plant tolerance to Fe deficiency through their interaction with and phosphorylation of IRT1 at the Ser149 residue, providing evidence that Ca^2+^ signaling directly mediates Fe absorption by regulating IRT1 (Wang et al., 2023). In addition, involvement of CBL1/9–CIPK23 in the process of Fe deficiency has been identified, and the *cipk23* mutant exhibits sensitivity to Fe deficiency because of reduced ferric chelate reductase activity (Tian et al., 2016). When Fe deficiency was accompanied by increased availability of non-Fe metals in the soil, CIPK23 phosphorylated IRT1 at the S/T residues to facilitate recruitment of the E3 ubiquitin (Ub) ligase IRT1 degradation factor 1 (IDF1) for efficient endosomal sorting and subsequent degradation, thereby preventing IRT1 from transporting non-Fe metals such as Zn, Cu, and Mn (Dubeaux et al., 2018). CPK21/23 promote Fe absorption by phosphorylating IRT1 under low-Fe conditions, whereas phosphorylation of IRT1 by CIPK23 promotes its efficient sorting and subsequent degradation under low-Fe and high non-Fe metal stress, preventing plants from absorbing excess non-Fe metals while compromising Fe absorption. These two processes achieve precise regulation of plant metal stability by regulating IRT1. In addition, under Fe deficiency, activation of CIPK11, mediated by Ca^2+^-triggered CBL1/9, and subsequent phosphorylation of the basic-helix-loop-helix transcription factor fer-like Fe deficiency-induced transcription factor (FIT) convert inactive FIT into active FIT, further contributing to plant adaptation to Fe deficiency (Gratz et al., 2019; Figure 2).

### Ca^2+^ signaling maintains Mn transport and homeostasis

Mn is an important cofactor of more than 30 enzymes, an essential element in the metalloenzyme cluster of the photosystem II oxygen-evolving complex, and a requirement for multiple steps in the biosynthesis of carbohydrates, lipids, and lignin in plants (Schmidt et al., 2016; Alejandro et al., 2020; Xie et al., 2023). It is therefore important to maintain plant Mn homeostasis through Mn uptake and transport.

A series of recent studies has elucidated the regulatory mechanism of Ca^2+^ signaling in Mn uptake and transport in plants. Mn deficiency induces a pattern of long-lasting multicellular Ca^2+^ oscillations, with maximum concentrations spatially confined to specific cell groups in the root elongation zone. CPK21 and CPK23 interact with and phosphorylate the PM-localized, high-affinity Mn transporter NATURAL RESISTANCE-ASSOCIATED MACROPHAGE PROTEIN 1 (NRAMP1) at the Thr498 residue, enhancing the transport activity of NRAMP1 and facilitating Mn^2+^ absorption under conditions of Mn depletion (Fu et al., 2022; Huang, 2022; Figure 2). High-Mn stress also leads to an increase in cytoplasmic Ca^2+^ concentration and the generation of distinct Ca^2+^ signals, which differ in time, frequency, and amplitude from those observed under low-Mn stress (Zhang et al., 2021; Fu et al., 2022). The CBL1/9–CIPK23 complex senses Ca^2+^ signals and phosphorylates NRAMP1 at the Ser20/22 residues, promoting clathrin-mediated endocytosis of NRAMP1 and reducing Mn absorption by plants under high-Mn stress (Zhang et al., 2023). Metal tolerance protein 8 (MTP8), a member of the cation diffusion facilitator (CDF) family, functions as a vacuolar Mn/Fe transporter and plays an important role in Mn detoxification in plants (Eroglu et al., 2016, 2017). In the cytoplasm, four activated Ca sensors (CPK4, CPK5, CPK6, and CPK11) interact with MTP8 and phosphorylate its Ser31/32 residues to facilitate transport of excess Mn^2+^ to the vacuoles for segregation, ultimately improving tolerance to Mn toxicity (Zhang et al., 2021). Intriguingly, after prolonged exposure to Mn toxicity, other Ca sensors, CBL2/3, recruit CIPK3/9/26 to form a complex that phosphorylates MTP8 primarily at Ser35, ultimately inhibiting its activity and acting as a braking mechanism (Ju et al., 2022) (Figure 2). These processes synergistically regulate Mn homeostasis in plants under fluctuating environmental Mn conditions.

Although the molecular mechanisms underlying the Ca^2+^ signals that regulate plant Mn homeostasis have been partially elucidated, the mechanisms by which Ca^2+^ signals are generated and regulate Mn homeostasis in other subcellular structures, such as the Golgi apparatus and ER, remain unclear. Therefore, future work will focus on elucidating mechanisms of Ca^2+^ signal generation and regulation in these subcellular structures under Mn stress. In addition, plants often encounter multiple, simultaneous element stresses in the soil under natural conditions. For example, when plants are subjected to both low Fe and low Mn stress, the resulting Ca^2+^ signals differ in duration, amplitude, and frequency. CPK21/23 detect these different Ca^2+^ signals and then phosphorylate and modify different substrates, such as IRT1 or NRAMP1, to transmit the Ca^2+^ signals. However, how plants recognize and accurately transmit signals to produce specific responses in the face of complex environmental changes remains a focal point and a challenge for future research.

### Role of Ca^2+^ signaling in Cu metabolism

Cu is an essential micronutrient for plant development and a cofactor for various enzymes (Burkhead et al., 2009). In *Ulva compressa* (a marine alga), excessive Cu induces Ca^2+^ release from the ER, and the ryanodine-sensitive and IP3-sensitive Ca channels in the ER are activated in response to excess Cu (González et al., 2010). Cu-induced activation of L-type voltage-dependent Ca channels and transient receptor potential (TRP) channels leads to intracellular Ca^2+^ release, which requires extracellular Ca entry (González et al., 2012b; Gómez et al., 2015). Increases in Ca^2+^ induced the activation of defense genes via CaMs and CDPKs under conditions of Cu excess (González et al., 2012a). In addition, activation of CaMs and CDPKs leads to Cu entry and membrane depolarization (Gómez et al., 2015). To date, TRP has been observed in mammals, insects, nematodes, and macroalgae but not in plants (González et al., 2018). Further investigation is needed to determine whether similar mechanisms exist in plants and whether Ca^2+^ sensors, including CBL–CIPK and CPK, directly regulate Cu transporters.

### B starvation causes Ca^2+^ influx

B is an essential element for plant growth, and B deficiency induces various physiological and metabolic alterations in plant cells (Brdar-Jokanović, 2020; Dong et al., 2022b). Cells subjected to short-term B deprivation show increased Ca^2+^ uptake, likely via Ca^2+^ channels (Koshiba et al., 2010). B starvation enhances cytosolic Ca^2+^ levels and expression of CNGC19, ACA and CAX efflux, and Ca^2+^ sensor genes in *Arabidopsis* roots (Quiles-Pando et al., 2013; González-Fontes et al., 2014). Upon B resupply, Ca^2+^ levels are restored, and CAX3 plays a major role in maintaining Ca^2+^ homeostasis (Quiles-Pando et al., 2019). It would be interesting to explore the direct involvement of Ca^2+^ sensors in regulation of B transporters.

#### Ca^2+^ signaling helps to regulate plant absorption of beneficial elements and detoxification of toxic elements

As attention to crop quality and human health has increased, the study of beneficial elements has become important. There is a class of elements that promote plant growth at suitable concentrations, but are not necessary for plant growth, such as Si, Na, Co, and Se (Pilon-Smits et al., 2009; Vatansever et al., 2017). Studying the growth regulation of beneficial elements in plants can not only improve plant yield and quality but also increase human intake of beneficial elements through food.

As a direct source of mineral nutrients for plants, the soil may also contain heavy metal elements that are toxic to plants. To attenuate the toxic effects of these metals, plants must develop interpretative mechanisms. Evidence suggests that the Ca–CaM pathway is involved in the response to Cd, Pb, Cr(VI), As, and Al toxicity (Tang et al., 2023).

### Ca^2+^ signaling is involved in Na homeostasis

Na in the soil is an important nutrient for plant growth and development. However, at high concentrations, it disturbs and inhibits various physiological processes and plant growth (Zhu, 2016).

As an Na^+^ sensor, glycosyl inositol phosphorylceramide senses high salinity and triggers Ca^2+^ influx, producing a rapid and transient increase in cytosolic Ca^2+^ levels (Jiang et al., 2019). Salinity stress triggers several responses, including Ca^2+^ oscillations, which play a multifaceted role in eliminating detrimental effects (Schmöckel et al., 2015). In addition, FERONIA, a PM local receptor kinase, plays a key role in maintaining plant cell wall structure under salinity stress and is mainly associated with Ca^2+^ signaling cascades by regulating Ca^2+^ channel activity (Okubo-Kurihara et al., 2016; Feng et al., 2018). Furthermore, Na^+^ influx into cells can be sensed by non-selective cation channels (Wu, 2018), and elevation of cytosolic Ca^2+^ content is also regulated by two-pore channels, Ca^2+^-ATPases, and CAXs (Wilkins et al., 2016). These regulatory modules enable plants to rapidly sense and produce specific Ca^2+^ signals under salt stress.

Under normal physiological conditions, plants generally maintain low Na^+^ concentrations ranging from 1–10 mM (Binzel et al., 1988). Studies have shown that *cngc3* null mutations result in decreased salt tolerance, whereas knockout of *CNGC10* leads to increased tolerance of salt stress. This suggests that CNGC3 and CNGC10, which are located on the PM, function as channels for Na^+^ influx in *Arabidopsis* (Gobert et al., 2006; Guo et al., 2008; Jin et al., 2015).

**Figure 3:**
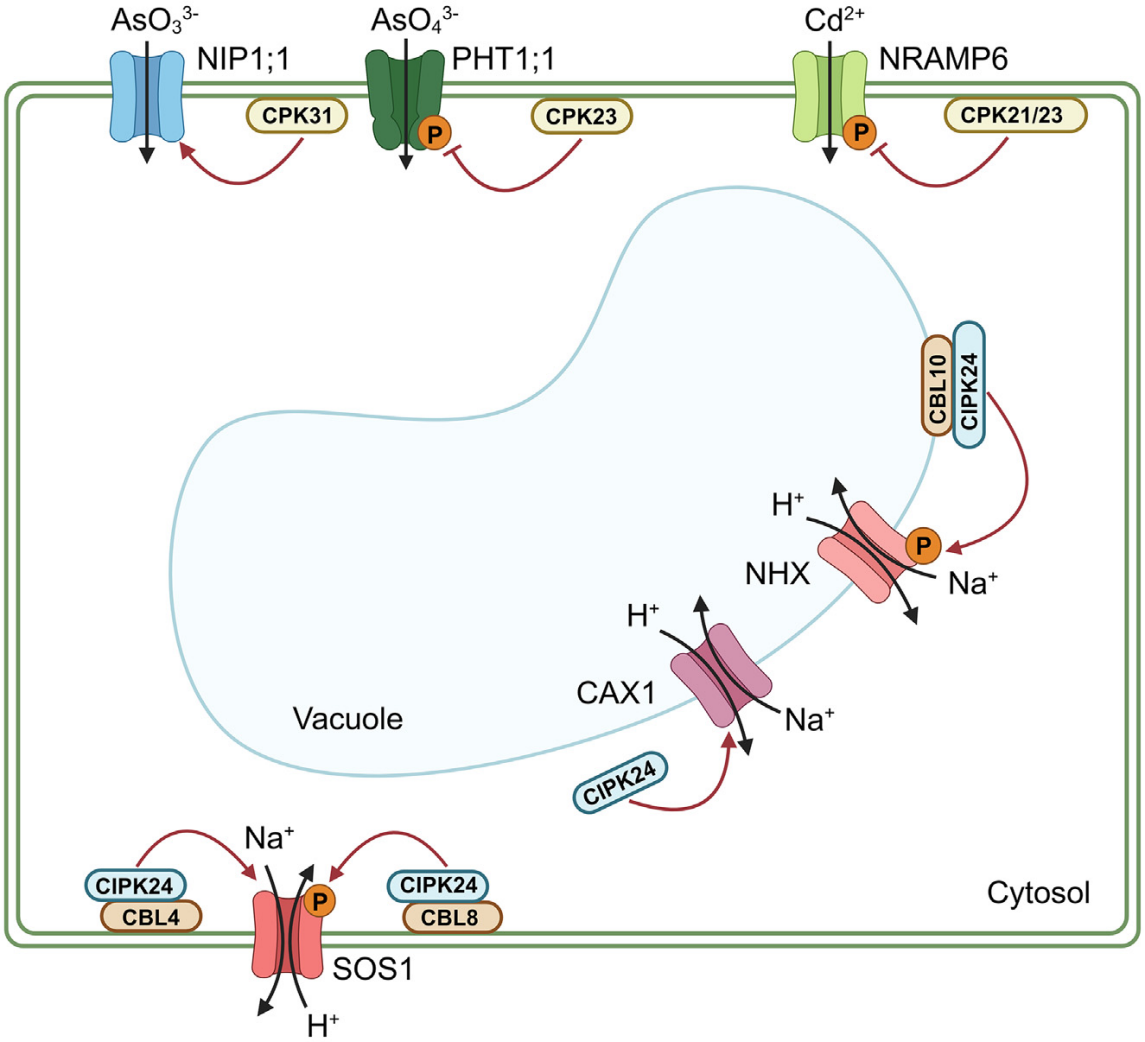
Ca^2+^ signaling regulates absorption of beneficial elements and detoxification of toxic elements in plants. In the classic SOS pathway, Ca^2+^ signals are sensed by CBL4/SOS3, which binds to and activates the kinase activity of CIPK24/SOS2. CIPK24, in turn, phosphorylates and enhances the Na^+^ efflux activity of SOS1. Under high-salinity stress, CBL8 in *Arabidopsis* can also activate CIPK24, further enhancing the function of the SOS pathway in Na^+^ efflux. In addition, tonoplast-localized CBL10 is required for salt tolerance and activates an NHX together with CIPK24, facilitating compartmentalization of Na^+^ in the vacuole. Apart from its involvement in the SOS pathway, CIPK24 also plays a role in regulating the vacuolar H^+^/Ca^2+^ antiporter CAX1. Regarding arsenic (As) stress responses, CPK31 has been shown to interact with the As(III) transporter NIP1;1, influencing As(III) uptake and tolerance. In addition, CPK23 phosphorylates PHT1;1 and regulates its subcellular localization under As(V) stress. For cadmium stress responses, CPK21/23 phosphorylate NRAMP6, inhibiting its Cd transport activity and ultimately enhancing Cd tolerance. SOS, salt overly sensitive; NHX, Na^+^/H^+^ antiporter; CAX, H^+^/Ca^2+^ antiporter; NIP1;1, nodulin 26-like intrinsic protein 1;1; PHT1;1, Pi transporter 1;1; NRAMP6, natural resistance-associated macrophage protein 6.

Na^+^ is an abundant element in soils and soil solutions, and salinity limits plant growth and impairs agricultural productivity. Na^+^/H^+^ antiporters (NHXs) such as PM salt overly sensitive 1 (SOS1) and vacuolar NHX1 have been shown to enhance salt tolerance in plants (Khan et al., 2015). The SOS pathway is the classic mechanism by which plants export excess Na^+^. NHX SOS1 is localized in the PM and closely associated with the CBL–CIPK network (Luan et al., 2009). In the classic SOS pathway, Ca^2+^ signals are sensed by CBL4 (SOS3), which binds to and activates the kinase activity of CIPK24 (SOS2), and SOS1 is phosphorylated by CIPK24, which enhances Na^+^ efflux (Liu and Zhu, 1998; Liu et al., 2000; Shi et al., 2000; Qiu et al., 2002; Figure 3). In addition to the classic pathway, CBL8 in *Arabidopsis* can activate CIPK24 under high salinity stress, further enhancing the function of the SOS pathway in Na^+^ efflux (Steinhorst et al., 2022). Recently, a study found that phosphatidic acid binds to the Lys57 residue in CIPK24, which activates SOS1 to promote Na^+^ efflux under salt stress. Interestingly, phosphatidic acid promotes phosphorylation of SOS3-like Ca-binding protein 8 (SCaBP8/CBL10) by CIPK24 under salt stress, which attenuates the SCaBP8-mediated inhibition of AKT1 (Li et al., 2023a).

As an independent component of kinase activity, CIPK24 has also been found to regulate the vacuolar H^+^/Ca^2+^ antiporter CAX1 (Cheng et al., 2004). CBL10 is required for salt tolerance, presumably by activating a vacuolar NHX together with CIPK24, enabling compartmentalization of Na^+^ into the vacuole (Kim et al., 2007). However, SOS2 has been shown to phosphorylate CBL10 to stabilize the CBL10–SOS2 complex and enhance PM Na^+^/H^+^ exchange activity to promote Na^+^ efflux (Quan et al., 2007; Lin et al., 2009; Figure 3). In conclusion, the Ca^2+^–CBL–CIPK signaling pathway plays a significant role in regulation of salt stress, and it remains of great interest to explore the functions and molecular mechanisms of other Ca^2+^ sensors and Ca^2+^ channels under salt stress.

### Ca^2+^ signaling is involved in plant heavy metal detoxification

Cd is a nonessential metal that can be transported into plants through Ca^2+^ channels, causing Cd toxicity (Perfus-Barbeoch et al., 2002; Haider et al., 2021). Fluorescence imaging with the Ca^2+^-specific fluorescent probe 4-AM showed that Ca^2+^ signals were stimulated by exogenous Cd in duckweed (*Lemna turionifera)* rhizoids (Yang et al., 2020), and transgenic duckweed expressing a Ca^2+^-sensing fluorescent sensor GCaMP3 showed a Ca^2+^ signal response during Cd stress (Ren et al., 2022). Likewise, transgenic *Arabidopsis* expressing GCaMP6, a novel ultrasensitive Ca^2+^ sensor, exhibited obvious Ca^2+^ signals in the root meristematic zone under high-Cd stress. Furthermore, CPK21/23 phosphorylate NRAMP6 primarily at Ser489 and Thr505 to inhibit its Cd transport activity, thereby improving plant Cd tolerance (Zhang et al., 2022; Figure 3).

As is a metalloid with heavy-metal properties that is ubiquitous in many environments (Chen et al., 2019). Dietary intake of arsenate-contaminated plant-derived food represents a major fraction of potentially health-threatening human exposure to As. Recently, As(V) stress was shown to induce a significant Ca^2+^ signal in *Arabidopsis* roots that appeared in the root maturation zone and gradually increased in the middle column zone. CPK23 phosphorylates Pi transporter 1;1 (PHT1;1) at Ser514 and regulates PHT1;1 subcellular localization under As(V) stress (Liu et al., 2023), and CPK activity is markedly enhanced under As(V) stress in rice (Huang et al., 2012). In this regard, CPK31 has been found to interact with nodulin 26-like intrinsic protein 1;1 (NIP1;1) and determine As(III) uptake and tolerance in *Arabidopsis* (Ji et al., 2017; Figure 3).

Significant progress has been made in understanding Ca^2+^ signaling in heavy metal regulation, and it is clear that Ca^2+^ signaling is involved in heavy metal detoxification in plants. Nonetheless, there are still gaps in our understanding of the involvement of Ca^2+^ signaling in heavy metal toxicity.

#### Regulation of Ca^2+^ signaling in response to other essential mineral nutrients, beneficial elements, and toxic elements

S, Zn, Cl, Mo, and Ni are essential mineral nutrients for plants (Maathuis and Diatloff, 2013), and many studies have confirmed that Si, Co, and Se are beneficial for plant growth and development (Gui et al., 2022; Hu et al., 2021; Wiese et al., 2007). Under natural conditions, heavy metals such as Al, Pb, and Cr in soil can significantly hinder plant growth and disrupt normal development of roots, stems, and other tissues (Yadav et al., 2021). However, little has been reported about the involvement of Ca^2+^ signaling in regulating the absorption and transport of these elements.

Exogenous supplementation with Ca^2+^ and NO efficiently mitigates Ni toxicity and regulates growth and development of the cyanobacterium *Nostoc muscorum ATCC 27893*, implying a signaling role for Ca^2+^ and NO in response to Ni stress (Verma et al., 2021). In addition, some effects of Ca^2+^ on Ni tolerance have been reported to be related to triggering of Ca^2+^ signaling in *Cucurbita pepo* L. (Valivand and Amooaghaie, 2021). Under Zn deficiency, transcript levels of some genes in the CAM, CML, CPK, and CBL–CIPK families of Ca^2+^ sensors change when Zn is resupplied, suggesting that these Ca^2+^ sensors may respond to Zn deficiency (Arsova et al., 2019). Previous studies have shown that CML24 regulates ALMT1-dependent resistance to Al (Zhu et al., 2022). A CaM-binding protein (NtCBP4) of tobacco decreases Ni accumulation and increases Pb accumulation (Arazi et al., 1999).

The mechanism by which Ca^2+^ signaling participates in regulation of these essential mineral nutrients and beneficial elements is not well understood, and this part of the regulatory network requires further analysis. The functions of Ca^2+^ channels in generation of specific Ca^2+^ signals during S, Zn, Cl, Mo, and Ni stress, as well as the functions of CBL–CIPK modules and CPKs in absorption and transport of these elements, will be a major focus of research on Ca^2+^ signal influence on essential mineral nutrient regulation. A thorough understanding of how Ca^2+^ channels generate specific Ca^2+^ signals during absorption of beneficial elements and detoxification of different metals or metalloids, and how these signals are transmitted through Ca^2+^ sensors, will greatly enhance our understanding of the mechanisms that underlie the involvement of Ca^2+^ signals in absorption of beneficial elements and heavy metal toxicity.

### Concluding remarks and future prospects

#### Ca^2+^ signal generation in response to different elements

A considerable number of studies have indicated that Ca^2+^ signaling plays a crucial role in regulating plant nutrient uptake, nutrient transport, and various nutrient stresses. It has been observed that different nutrient element stresses can induce changes in cytoplasmic Ca^2+^ concentration, thus generating specific Ca^2+^ signals. These nutrient elements encompass macronutrients such as N, P, and K; micronutrients including Fe and Mn; and beneficial and toxic elements such as Na, Cd, and As (Tian et al., 2016; Riveras et al., 2015; Jiang et al., 2019; Zhang et al., 2021, 2022; Fu et al., 2022; Ying and Scheible, 2022; Liu et al., 2023). Although Ca^2+^ signals primarily manifest in the roots under nutrient stress, their occurrence and characteristics in shoot tissues have received limited attention to date. Furthermore, it will be important to elucidate the encoding and decoding mechanisms for specific Ca^2+^ signals under different nutrient element stresses, as they are inherently stimulus-specific in terms of their magnitude, location, and duration. These stimulus-induced changes in cytoplasmic Ca^2+^ concentration produce unique spatial and temporal patterns known as Ca^2+^ signatures (Tian et al., 2020).

Taking Mn as an example, Mn depletion triggers spatiotemporally distinct, long-lasting multicellular Ca^2+^ oscillations in *Arabidopsis* roots. These Ca^2+^ signals initially emerge in individual cells before spreading intercellularly, gradually intensifying, and finally transforming into higher-order multicellular oscillations. In response to high Mn exposure, a transient Ca^2+^ signal begins to rise approximately 17 min after the initiation of stress, steadily reaching its peak at around 18 min, and declining thereafter (Fu et al., 2022). Further exploration of similar cases under different nutrient stresses will provide valuable insights into the specific mechanisms that underlie Ca^2+^ signal generation, propagation, and decoding. Additional insight into these processes will make a significant contribution to our understanding of plant responses to nutrient stresses and heavy metal detoxification.

### Intracellular Ca^2+^ imaging in plant abiotic stress research

Ca^2+^ indicators are essential for studying the concentrations of Ca^2+^ in various cells or tissues and are considered indispensable tools in this field. They can be broadly categorized into two types based on their fluorescence spectra, Ca^2+^ affinity, and chemical characteristics: chemical indicators and genetically encoded Ca indicators (GECIs).

Chemical indicators, such as fura-2, indo-1, fluo-3, and fluo-4, have long been used as traditional tools for detecting Ca^2+^ levels in the cytoplasm. These indicators rely on the binding of Ca^2+^ to specific fluorescent molecules, enabling measurement of Ca^2+^ concentrations in real time (Paredes et al., 2008). On the other hand, GECIs represent a novel class of Ca^2+^ indicators that have emerged with advances in genetic engineering technology. They enable long-term and real-time monitoring of Ca^2+^ levels *in vivo*. GECIs can also provide insights into changes in Ca^2+^ in specific subcellular structures through the use of organelle-specific localization signals. These remarkable features of GECIs have contributed to their widespread use for *in vivo* Ca imaging experiments. Precise measurement and visualization of Ca^2+^ dynamics using these indicators have become essential tools for understanding the complex and diverse functions of Ca^2+^ signaling in living systems.

According to the principle of luminescence, GECIs can be divided into two categories: GECIs based on a single fluorescent protein and GECIs composed of fluorescent protein pairs that undergo fluorescence resonance energy transfer (Miyawaki et al., 1997; Nakai et al., 2001).

In plant research, Yellow Cameleon 3.6 is a commonly used fluorescence resonance energy transfer–based fluorescent Ca^2+^ indicator. It has been used extensively to monitor plant Ca^2+^ kinetics and investigate the relationships between Ca^2+^ signaling and various physiological processes such as root hair growth, pollen tube tip growth, and stomatal response (Monshausen et al., 2008; Swanson and Gilroy, 2013; Thor and Peiter, 2014; Zhang et al., 2020). Another popular Ca^2+^ indicator in plants is GCaMP, which is based on a single fluorescent protein. GCaMP6, in particular, has shown high sensitivity and is suitable for detecting low-frequency signals (Nakai et al., 2001; Chen et al., 2013). Advances have also been made in producing multifunctional Ca indicators based on GCaMP. For instance, the ratiometric Ca^2+^ indicator R-GECO1-mTurquoise and MatryoshCaMP6s have proven to be effective tools for mapping absolute Ca^2+^ concentration changes under different elemental stresses (Ast et al., 2017; Waadt et al., 2017). The Ca^2+^ sensor GCaMP6f-mCherry combines the superior dynamic range and temporal accuracy of GCaMP6f with ratiometric data acquisition via mCherry emission monitoring standardization. This method has been used to detect Ca^2+^ signals under Mn deficiency, demonstrating its potential for investigating trace element–induced Ca^2+^ signaling (Fu et al., 2022). These advances and applications help to facilitate the detection of Ca^2+^ signals under nutrient stress, enabling further research in this area.

#### Role of the Ca^2+^ signal regulatory network in regulation of mineral elements

In previous reports, the CBL1/9–CIPK23 module has been shown to participate in regulating the absorption and transport of nutrients such as N, K, and Fe. Similarly, the CBL2/3–CIPK3/9/23/26 module is involved in regulation of Mn and Mg, and CPK21/23 have been found to play a role in absorption and transport of Mn, Fe, As, and Cd (Tang et al., 2020b; Ju et al., 2022; Fu et al., 2022; Wang et al., 2023; Zhang et al., 2022; Liu et al., 2023). An interesting question is how the same CBL–CIPK or CPK modules perceive and differentiate specific environmental stimuli, enabling them to accurately regulate different downstream effectors. Previous studies have shown that Ca^2+^ signals generated by plants under various stresses are distinct, exhibiting differences in timing, spatial distribution, and amplitude (Luan and Wang, 2021; Dong et al., 2022a). As a result, the same CBL–CIPK or CPK modules are thought to recognize specific Ca^2+^ signals and elicit unique responses to different stresses.

Ca^2+^ sensors are found in various locations in the plant cell, including the PM and VM (Sanyal et al., 2015). However, the synergistic regulation of Ca^2+^ signal transduction networks mediated by different plant Ca^2+^ sensors, particularly those in the PM, VM, or other membrane systems, remains to be further investigated. This is an area in which future research is needed to elucidate the intricate mechanisms that underlie coordination of Ca^2+^ signaling pathways in response to different stimuli.

Under natural conditions, the environment is characterized by variability and complexity, necessitating further investigation of the crosstalk among Ca^2+^ signal transduction networks under different nutrient stresses. One area of interest is the Ca^2+-binding^ affinity of different Ca^2+^ sensors. It is important to understand whether different Ca^2+^ sensors can perceive distinct ranges of Ca^2+^ concentrations to initiate specific signaling pathways. Overall, there are still significant gaps in our understanding of the temporal, spatial, and intensity changes in Ca^2+^ signal production under different stress conditions, as well as the differential responses of different Ca^2+^ channels to environmental signals. Moreover, the molecular mechanisms by which CaM and CML participate in different nutrient stress responses remain to be elucidated. In addition to further exploring the biological processes by which Ca^2+^ signaling regulates plant nutrient-stress responses, future research will also focus on using biotechnological applications to cultivate stress-tolerant crops.

## Funding

This work was supported by the 10.13039/501100001809National Natural Science Foundation of China (32222008 to C.W.) and the 10.13039/501100002858China Postdoctoral Science Foundation (2023M732883 to C.J.).

## Author contributions

All work was performed in collaboration. T.W. and C.W. wrote the original draft and prepared the figures. T.W., X.C., C.J., and C.W. designed and finalized the manuscript. The authors have read and agreed to the published version of the article.
